# Sea Cucumber Egg Oligopeptides Ameliorate Cognitive Impairments and Pathology of Alzheimer’s Disease Through Regulating HDAC3 and BDNF/NT3 via the Microbiota–Gut–Brain Axis

**DOI:** 10.3390/nu17142312

**Published:** 2025-07-14

**Authors:** Guifeng Zhang, Yanjie Dou, Huiwen Xie, Dan Pu, Longxing Wang, Renjun Wang, Xiaofei Han

**Affiliations:** 1 Key Laboratory of Saccharide and Lipid Metabolism Research in Liaoning Province, College of Life and Health of Dalian University, Dalian 116622, China; 2 Dalian Institute of Chemical Physics, Chinese Academy of Sciences, Dalian 116023, China

**Keywords:** Alzheimer’s disease, sea cucumber egg oligopeptides, cognitive dysfunction, gut microbiota, neuropathology

## Abstract

Background: Oligopeptides from sea cucumber eggs (SCEPs) are rarely studied for their neuroprotective effects. Methods: Therefore, we prepared SCEPs via simulated gastrointestinal digestion and then administered them to an Alzheimer’s disease (AD) mouse model via gavage. Behavior tests, gut–brain histopathology and fecal microbiota transplantation (FMT) experiments were conducted, and gut microbiota and metabolite short-chain fatty acids (SCFAs) were evaluated via 16sRNA gene sequencing and LC-MS. Results: The results showed that both the SCEP and FMT groups experienced improvements in the cognitive impairments of AD and showed reduced levels of Aβ, P-Tau, GFAP, and NFL in the brain, especially in the hippocampus. SCEP remodeled the gut microbiota, increasing the relative abundances of *Turicibacter* and *Lactobacillus* by 2.7- and 4.8-fold compared with the model at the genus level. In the SCEP and FMT treatments, four SCFA-producing bacteria obtained from gut microbiota profiling showed consistent trends, indicating that they may be involved in mediating the neuroprotective effects of SCEP. Mechanically, SCEP regulated the SCFA distribution in feces, blood, and the brain, greatly increased the content of SCFAs in the brain up to 2000 μg/mg, eased gut–brain barrier dysfunction, inhibited HDAC3 overexpression, and upregulated BDNF/NT3 levels. Conclusions: This study provides a promising candidate for preventing AD and a reference for applying SCEP.

## 1. Introduction

Alzheimer’s disease (AD) is a chronic neurodegenerative disease characterized by progressive cognitive dysfunction and behavioral impairment. Clinically, AD typically presents with memory loss, cognitive decline, mood changes, and declines in thinking and behavioral skills [1]. Two neuropathological hallmarks of the AD brain have been well recognized: β-amyloid plaques and tau neurofibrillary tangles [2]. Current treatments can only provide temporary symptomatic relief for AD patients [3]. In recent years, the incidence of AD has been increasing, bringing significant barriers and pressures to individual health and public welfare. Therefore, strategies to prevent the appearance of pathological features as early as possible are of great importance.

Marine organisms have been recognized as valuable repositories of bioactive compounds with important neuroprotective and cognitive enhancement potential. The sea cucumber is recognized for its rich composition of unsaturated fatty acids, bioactive peptides, saponins, and polysaccharides [4]. Among them, sea cucumber peptide has been found to have antioxidant, Angiotensin-I Converting Enzyme activity inhibition, and anti-aging activities [5]. Previous studies have suggested that sea cucumber peptide exhibits anti-aging effects in fruit flies by regulating microbiota and metabolic disorders [6]. Similarly, low-molecular-weight sea cucumber peptides of less than 3 kDa have been reported to protect against oxidative stress and regulate the cholinergic system in scopolamine-induced PC12 cells, as well as improve synaptic plasticity [7]. In addition, numerous studies highlight sea cucumber peptide intervention as a promising strategy to solve memory disorders and maintain brain function. Compared with synthetic drugs, peptides from food proteins have been found to be safer, milder, and more easily absorbed [8].

The increasing global demand for protein has pushed the recycling of marine by-products to the forefront of research and has been widely studied in the biomedical field [9]. Pepsin-dissolved collagens from tilapia skin and bovine collagen electrospun scaffolds have significant bioactivity and can accelerate wound healing rapidly and effectively in rat models [10]. Marine collagen is used for injectable dispersions, microparticles, nanospheres, and capsules in drug delivery systems, where fish, jellyfish, sponges, and other vertebrates are a rich source of collagen [11,12]. They are characterized by biocompatibility, biodegradability, low antigenicity, and cell adhesion [13]. Thus, seafood by-products have broad application prospects in the medical industry.

Sea cucumber eggs, often regarded as a by-product of sea cucumbers, are an important part of sea cucumber viscera. There is still limited in-depth processing and product form development research on the functions of sea cucumber eggs, and the added value of these resources is low. In fact, sea cucumber eggs have similar nutritional contents, such as protein and fatty acids, to sea cucumber body walls [14]. They contain various active ingredients such as trace elements and enzymes, making them highly nutritious. However, research on the neuroprotective effects of sea cucumber egg peptides is still rare.

In recent years, the relationship between the intestinal microbiome and AD has become a research hotspot. Interestingly, the gut microbiome is known as the “second brain” due to its role in various immune, neuronal, and endocrine responses [15]. Under physiological conditions, the intestinal microbiome can promote the digestion and absorption of nutrients, participate in the synthesis of vitamins, affect the activity of enzymes, and regulate the immune function of the body [16,17]. Studies have shown that the gut microbiome affects brain development and function by changing its composition, species, and metabolites through bidirectionally connecting the gut–brain axis [18]. Relevant reports have revealed that neurotransmitters and metabolites regulate various immune system pathways; affect memory, learning, movement, and behavior; and even cause neurodegenerative diseases [19,20]. For example, short-chain fatty acids (SCFAs)are key microbial mediators in the gut–brain axis, affecting brain physiology and cognitive function [21].

However, there are no reports on the neuroprotective effects of oligopeptides from sea cucumber eggs (SCEPs). Therefore, this study aimed to prepare oligopeptides from sea cucumber eggs via simulated gastrointestinal digestion and investigated the neuroprotective effects of SCEPs on AD model mice. Subsequently, the underlying regulatory mechanisms determined from the perspective of the microbiota–gut–brain axis were assessed via fecal microbiota transplantation (FMT). We propose that SCEPs may exert neuroprotective effects by reshaping the gut microbiota structure in AD model mice, enhancing the composition and distribution of intestinal microbiota and its metabolites, and subsequently activating relevant antioxidant pathways. Furthermore, FMT experiments support the potential of SCEP to modulate the gut microbiota structure. This study contributes to the high-value utilization of sea cucumber eggs and provides information for developing marine-derived peptides. This study may support the prevention of AD before it develops.

## 2. Materials and Methods

### 2.1. Experimental Approach

Six-week-old ICR male mice (SPF-grade, non-specific pathogen grade) were purchased from Liaoning Changsheng Biotechnology Co., Ltd., Benxi, China. The animal experiment was authorized by the Science and Technology Department of Liaoning Province, and the experimental animal license number was k2024-002. The mice were housed in a clean animal room with free access to water and food. The mice lived at a room temperature of 25 ± 1 °C, with a relative humidity of 50% ± 10%, and in a 12 h light/dark cycle. According to the resource equation method, E = N − k, the experimental design was divided into three groups (control, model, and SCEP groups), with E = 15. Through calculating this equation, the minimum sample size was set at *n* = 6 per group [22]. One week after the mice adapted to the environment, the experimental units were assigned randomly. Specifically, random sequences were generated through the sample function of R software (R 4.4.2 version), and 36 mice were divided into 6 groups. Before grouping, we determined that all mice were male, and their weights were between 20 g and 22 g. Except for the normal control group, the mice in the other experimental groups were intraperitoneally injected with 40 mg/kg AlCl_3_ and 100 mg/kg D-gal every day for 2 weeks, followed by intraperitoneal injection of 20 mg/kg AlCl_3_ and 100 mg/kg D-gal every day for 4 weeks. Mice in the SCEP group were treated with SCEP for 6 weeks. SCEP were prepared into a solution with normal saline at a ratio of 100 mg:100 μL. The dose was calculated according to the conversion formula of human and mouse, and the intragastric dose was 300 mg/kg/d. The daily intervention of mice was performed at a fixed time. In the second batch of experiments, the FMT_SCEP group mice were pretreated with antibiotics for 1 week and then administered fresh fecal samples, collected in advance, via gavage for 6 weeks. The specific experimental design and schedule are outlined in the Supplementary Materials (Tables S1 and S2).

### 2.2. Materials

Fresh sea cucumber eggs were purchased from the local market (Dalian, Liaoning Province, China). D-galactose (98%, D810318, Macklin, Shanghai, China), anhydrous aluminum chloride (99%, A800394, Macklin, Shanghai, China), neutral formalin fixed solution (10%, G2161, Solarbio, Beijing, China), and a BCA kit (P0012, Beyotime, Shanghai, China) were used.

### 2.3. Preparation of Sea Cucumber Egg Peptide

In this study, fresh sea cucumber intestinal tissues, including gonads and viscera, were thoroughly washed. Precisely 283.93 g of clean tissue was homogenized with 500 mL of distilled water and incubated for 1 h at 50–55 °C. The homogenate was lyophilized, ground into powder, and stored for later use. Then, 17.0 g of the lyophilized powder was extracted with 95% (*v*/*v*) ethanol in an ultrasonic bath for 30 min. It was then centrifuged at 10,000× *g* for 30 min at 4 °C; we discarded the supernatant, repeated the process 3–5 times, and collected the precipitate for lyophilization. We transferred 10.0 g of freeze-dried powder to a reaction vessel. We added gastric electrolyte solution (255 mL), porcine pepsin (59.5 mg, activity ≥3000 U/mg), and sodium acetate solution (0.51 mL). We then adjusted the pH to 2.0–3.0 via continuous stirring at 37 °C (80 rpm) using 1 mol/L HCl. We maintained these conditions for 1 h. Then, we added porcine trypsin (59.50 mg, lipase activity ≥30,000 U/g), porcine trypsin (17.0 mg), intestinal electrolyte solution (100 mL), 7% (*w*/*v*) porcine trypsin phosphate buffer (PBS, 50 mL, activity ≥2500 U/mg), and 4% (*w*/*v*) bile salt mixture to the gastric digestive fluid before immediately adjusting the pH to 7.0 using preheated (37 °C) 0.1 mol/L NaOH. We then performed intestinal digestion for 2 h at 37 °C with constant shaking (80 rpm). We immersed the reaction vessel in a 90 °C water bath for 5 min to inactivate enzymes. Next, we cooled the digests, performed centrifugation at 10,000× *g* for 15 min at 4 °C, and collected the supernatant for lyophilization. We then stored the resulting powder at −20 °C for further analysis. For animal experiments, researchers can scale up the system to ensure sufficient dosage.

### 2.4. Observation and Sampling of Mice

During the entire experiment, the mice’s coat color, mental and exercise status, water consumption, and food intake were observed regularly every day, and the mice’s weights were monitored and recorded every week. Feces were collected from mice following 48 h of SCEP withdrawal for use in fecal microbiota transplantation (FMT) experiments. Subsequently, behavioral assessments were conducted on the mice. Upon completing behavioral testing, the mice were subjected to a 12 h fasting period prior to euthanasia, after which blood and tissue samples were collected. The collected samples were either fixed or stored at −80 °C for subsequent analysis of relevant biological indicators.

### 2.5. Morris Water Maze Test

Experiments were conducted using a swimming pool (40 cm high × 100 cm diameter) and a hidden platform (10 cm diameter), where the hidden platform was located 1 cm below the surface of the water, and the swimming paths of the mice were recorded using an imaging system placed above the pool. In the localization navigation experiment, groups of mice were trained for 5 days (4 trials per day) to locate the hidden platform. During these 5 days of trials, mice were placed in the pool facing the wall, and the time that it took for mice to locate the hidden platform, i.e., the escape latency, was recorded. During training, if the mice still could not find the platform within 120 s, the experimenter was required to guide the mice to the platform and allow the mice to stay on the platform for 30 s, while the escape latency of the mice was recorded as 120 s. On day 6, the platform was removed, and each mouse was placed in a fixed position in Quadrant II, where the mice swam for 120 s in the pool without the platform. Mouse movements were tracked using a computerized tracking system (KW-MWM, Nanjing, Calvin Instrument Co., Ltd., Nanjing, China), and the number of times the mice crossed the platform and the time spent in the target quadrant were recorded.

### 2.6. Y-Maze Test

The Y-maze (Shenzhen Ruiwo Life Technology Co., Ltd., Shenzhen, China) free alternation test evaluated the spatial working memory of mice by recording their spontaneous alternation behavior. The Y-maze consisted of three equidistant long arms (a, b, c) and a central area. Each arm had a length of 30 cm, a width of 6 cm, and a height of 15 cm. The angle between each two arms was 120 degrees, and the whole apparatus had a “Y” shape. The mice were placed at the end of any arm of the maze, without food-induced stimulation, and allowed to explore freely for 5 min. The direction of the exploration arm was recorded every time, and the continuous entry into three different arms (such as abc, acb, bac, bca, cab, and cba) was recorded as a correct alternation behavior. The order of each mouse entering the arm, the number of correct alternations, and the total number of times entering the arm were recorded.

The spontaneous alternation rate was as follows: (%) = [number of correct alternation reactions/(total number of arm insertions − 2)] × 100%.

### 2.7. Individual Nesting Test

Individual nesting experiments were carried out. To adapt them to a solitary environment, the mice used were first placed in a single cage for 48 h before the experiment. We replaced clean corncob bedding within 48 h and spread the bedding 1 cm at the bottom of the rat cage. At 6 pm, a square, clean, and odorless cotton piece with a side length of 5 cm was selected as the nesting material and placed in the middle of the rat cage with tweezers. Animals had adequate access to food and water throughout the testing period. Mice were scored for their nesting ability at 12, 24, and 48 h after the start of the experiment. Nest scoring was performed by three independent researchers who were unaware of the mice’s grouping, etc. The score was set based on the Supporting Materials (Table S3).

### 2.8. 16S Ribosomal RNA Gene Sequencing Analysis

Fresh mouse fecal samples were collected in sterile tubes, immediately frozen in liquid nitrogen, and then stored at −80 °C for use. According to the characteristics of the amplified 16S region, a small fragment library was constructed, and double-end sequencing was performed using the Illumina NovaSeq sequencing platform. After read splicing and filtering, operational taxonomic units (OTUs) with 97% sequence similarity and amplicon sequence variations (ASVs) with 100% sequence similarity were clustered, and high-quality reads were selected for bioinformatics analysis. Alpha diversity was calculated based on the ACE, Chao1, Shannon, and Simpson indices. Beta diversity analysis was used to assess structural changes in microbial communities between experimental groups using the Unweighted UniFrac distance measure and visualized via principal coordinate analysis. Linear discriminant analysis and heat map methods were used to detect differentially enriched taxa among different taxa at different levels. Quantitative Insights into Microbiological Ecology (QIME, v1.9.0, http://QIIME.org/, accessed on 30 December 2024) and R package 3.5.1 (https://www.R-project.org/, accessed on 30 December 2024) were used to amplify the 16S rRNA gene’s V4 to V5 region to analyze bacterial taxa in each stool sample.

### 2.9. Hematoxylin–Eosin Staining

Immediately after euthanasia, mouse brain and intestinal tissues were taken and fixed in a neutral formalin solution for 48 h, followed by dehydration, embedding, and sectioning. Hematoxylin and eosin dyes were used as directed. Finally, microscopic examination, image acquisition, and analysis were performed.

### 2.10. Western Blotting

After dissecting the mice, the obtained tissues were weighed, cut into small pieces, and added with a 10-fold volume of tissue lysate. The supernatant was collected after ultrasonication for 10 min. Total protein quantification (BCA method), protein denaturation, electrophoresis, membrane transfer, blocking, and primary antibody incubation at 4 °C overnight were performed, followed by washing the membrane and adding secondary antibody incubation for 1–2 h. ImageJ software (https://imagej.net/software/imagej/, accessed on 30 December 2024)was used to analyze and calculate the relative expression of target proteins in each group. Aβ_1_**_–_**_42_, P-Tau, GFAP, NF-L, MCT-1, HDAC3, occludin, and Claudin5 were purchased from Bioss, Beijing, China.

### 2.11. Nissl Staining

Nissl staining of fixed mouse brain tissue was performed using Nissl staining solution (C0117, Beyotime, Shanghai, China) according to the manufacturer’s guidelines. Subsequently, Nissl-positive cells in the cortex and hippocampal CA1 and CA3 regions were observed and photographed under a microscope (Nikon Corporation, Nikon Eclipse E100, Tokyo, Japen).

### 2.12. Immunohistochemistry and Immunofluorescence

We took a mouse tissue sample, quickly placed it into a fixing solution with a volume greater than 20 times the tissue volume, fully immersed it for 48 h, and then dried the tissue, made it transparent, and subjected it to wax immersion to allow the paraffin to uniformly penetrate the tissue. Then, the tissue block was cooled and solidified and cut into 3 μm slices. Sections were fixed on glass slides (SuperFrost Plus, Gerhard Menzel GmbH, Brunswick, Germany) and dewaxed at graded concentrations of xylene and ethanol. The slides were treated in TRS solution in a microwave oven for 30 min (2 × 6 min 360 W, 2 × 5 min 180 W, 2 × 4 min 90 W) for antigen retrieval. After cooling at room temperature for 30 min, they were transferred to 0.3% hydrogen peroxide in methanol to block endogenous peroxidase activity. Sections were washed with Tris-buffered saline and incubated with primary antibodies against ZO-1 (1:500, Servicebio, Wuhan, China), and HDAC3 (Servicebio, China). Biotinylated or luciferase secondary anti-rabbit antibodies (Servicebio, Wuhan, China) were used at a dilution of 1:200. Detection was performed using the ABC peroxidase system. The DAB solution is a peroxidase substrate.

### 2.13. Enzyme-Linked Immunosorbent Assay (ELISA)

Commercial kits (Animalunion Bio, Shanghai, China) were used to quantify NT3 in tissues.

### 2.14. Fecal Microbiota Transplantation Experiment

FMT into the recipient mice was performed as reported in [23]. In brief, fresh feces were collected from SCEP mice at 7:00 am every day, placed in preoxygenated sterile centrifuge tubes, and added with sterile PBS at a ratio of 100 mg:1 mL. The feces were homogenized completely with a vortex and then centrifuged (4 °C, 3000 r) for 10 min, and the supernatant was collected from the recipient mice via gavage (0.1 mL/10 g). The fecal suspension was prepared on the spot to ensure that fecal transplantation was completed within 2 h after collection. Before FMT, mice received an antibiotic cocktail intervention continuously for one week, namely gentamicin sulfate (5 mg/kg), furotoxin enteric-coated tablets (25 mg/kg), metronidazole tablets (100 mg/kg), and fluconazole tablets (1 mg/kg), with these components being prepared together for gavage administration, while benzylpenicillin (1 g/L) was added to water for drinking.

### 2.15. Quantification of Short-Chain Fatty Acids

The processing method of plasma, brain tissue, and feces samples, as defined in reference [24], was improved. Simply put, 150 μL of plasma solution was thoroughly mixed with acetonitrile solution and stored at 4 °C for 1 h. After centrifugation at 8000 r/min for 10 min at 4 °C, the supernatant was collected, passed through a 0.22 μm filter membrane, and stored for later use. We placed a 100 mg brain tissue sample in 10 samples of absolute ethanol solution, which were sonicated for 5 min under an ice bath. After centrifugation at 12,000 r/min at 4 °C for 15 min, the supernatant was collected, passed through 0.22 μm filter membrane, and stored for later use. A 100 mg feces sample was placed in a 10-fold volume ethanol solution and sonicated under an ice bath for 15 min. After centrifugation at 12,000 r/min at 4 °C for 15 min, the supernatant was collected and passed through a 0.22 um filter membrane for testing.

SCFAs were detected and quantified as described in [24]. An LC-MS/MS system equipped with a 1290 HPLC instrument (Agilent 1290, Agilent Technologies, Santa Clara, CA, USA), a QTRAP 5500 (AB Sciex, Waltham, MA, USA), and a reversed-phase column (Pursuit 5 C18 150 × 2.0 mm, Agilent Technologies, Santa Clara, CA, USA) for the LC-MS/MS system. The data were analyzed using Analyst 1.5.2 software (AB Sciex, Waltham, MA, USA), and the peak areas were normalized using an added internal standard.

### 2.16. Statistical Analysis

All experimental data were statistically analyzed using GraphPad Prism 8.5 software (GraphPad Software, San Diego, CA, USA). The results were reported as mean ± standard error (x ± SEM). Statistics were performed using Tukey’s Multiple Comparison Test or a t-test using One-way ANOVA. *p* < 0.05 was considered to be statistically different. In the statistical analysis, the data points in the following situations were excluded. In the event of technical errors, it was clear that the data were invalid due to instrument failure or operational mistakes (such as sampling errors, recording errors). Sample loss/contamination: due to improper sample processing (such as centrifugal rupture, contamination, etc.), valid measurement values could not be obtained. Extreme outliers: we used predefined statistical methods to identify and exclude extreme outliers (e.g., Grubbs’ test, set *p* < 0.01), or the definition exceeded the mean by ±3 SD.

## 3. Results

### 3.1. Effects of Gavage Administration of SCEPs on Cognitive and Emotional Deficits in the AD Mouse Model

The SCEPs prepared in our group with a low molecular weight of around 3000 Da, as determined via SDS-PAGE, were oligopeptides. The total protein content of the SCEP was 106 mg/g, as detected via the BCA method. The in vitro antioxidant activity of SCEP was investigated. SCEP treated via simulated gastrointestinal digestion had stronger antioxidant capacity in vitro than undigested egg peptides.

**Figure 1:**
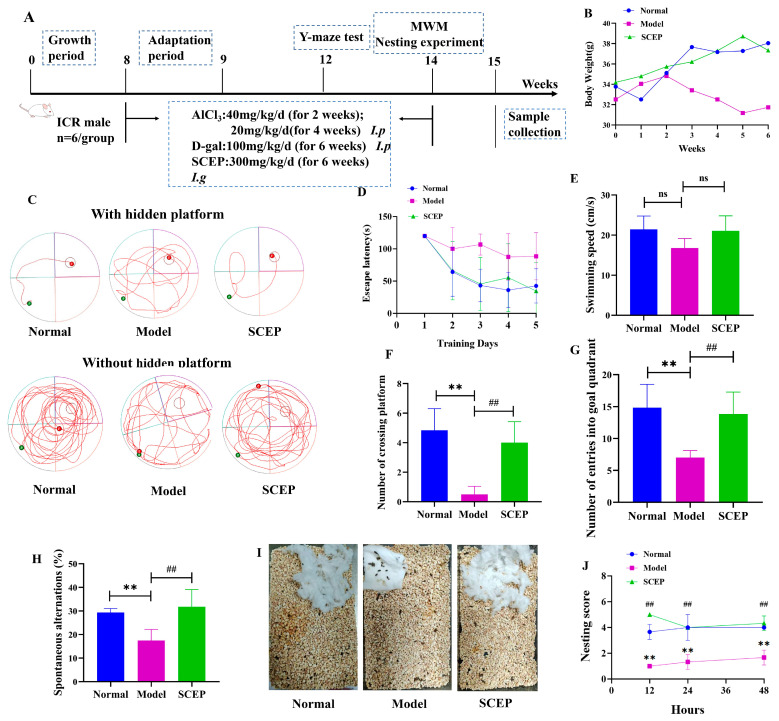
Effects of gavage administration of SCEP on behavioral impairments and cognitive deficits in AD mouse model. (**A**) Flow chart of the behavior experiment. (**B**) Body weight of mice. (**C**) Representative traces in MWM tests (green dot: starting position; red dot: end position). (**D**) Escape latency during training. (**E**) Average swimming speed. (**F**) The number of platform crossings. (**G**) Number of entries into the goal quadrant. (**H**) Y-maze experiment analysis. (**I**) Individual nesting test photographs and (**J**) analysis. Data were mean ± SEM (*n* = 6 per group). ns stands for no significant difference; **, ## *p* < 0.01 (* vs. normal group and # vs. model group, respectively).

The specific flow of behavioral experiments is shown in Figure 1A. As shown in Figure 1B, the body weight of the model mice showed a decreasing trend, while those of the other two groups, namely the SCEP group and the control group, showed increasing trends. During the period of the experiment, the average body weight of the model group mice was lower than that of the other two groups. The Morris water maze (MWM) is a classic method for evaluating animal spatial learning and memory abilities. During training, the model mice showed a long escape latency, presenting decreases in learning and memory ability with regard to the position of the platform. No significant difference in swimming speed was detected among each groups. Compared with the normal group, the model group successfully positioned the platform fewer times and stayed in the target quadrant for shorter times, showing impaired learning and spatial memory abilities. Compared with the model group, the SCEP group successfully positioned the platform more times (*p* < 0.01; d = 0.167) and stayed in the target quadrant for longer times (*p* < 0.01) (Figure 1C–G). The Y-maze is often used to evaluate the short-term spatial memory ability and cognitive function of animals. The results of the Y-maze showed that the spontaneous alternation rate of mice in the model group was lower than that in the normal group, indicating that the application of AlCl_3_/D-gal affected the learning and memory ability of mice. Excitingly, compared with the model group, SCEP intervention increased the spontaneous alternation rate of AD mice (*p* < 0.01; d = 0.8), as shown in Figure 1H. Nesting is a common daily behavior with a complex physiological basis that is controlled by many brain regions and correlates with hippocampal area function. We evaluated the improvement derived from the SCEP intervention on living ability and the function of the hippocampal area in AD mice via individual nesting experiments. The results showed that the model group showed obvious non-nesting behaviors within 48 h (the cotton sheets used in the experiment were scattered in the cage, with no obvious nests and no obvious bite marks), and the nesting scores were significantly lower than in the normal group (*p* < 0.01) (the cotton sheets were obviously torn and aggregated by the animals and piled up to form a deep nest), while the mice undergoing SCEP treatment started to build nests within 12 h and formed more complete nests within 24 h, showing higher nesting behavior scores than in the model group (*p* < 0.01) (Figure 1I,J). These results indicate that the gavage administration of SCEP could ameliorate learning and memory impairments, improving living ability and emotional deficits in AD model mice.

### 3.2. Effects of Gavage Administration of SCEP on the Pathology of the AD Mouse Model

**Figure 2:**
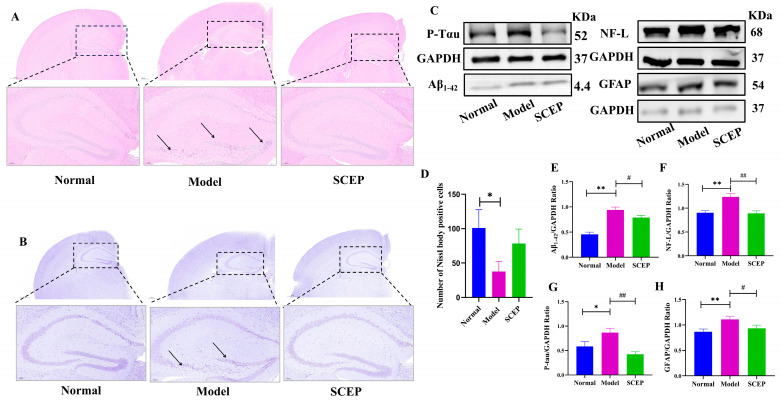
The effects of the gavage administration of SCEP on the pathology of the AD mouse model. (**A**) H&E staining (Black arrows represent neuronal pyknosis) (**B**) Nissl staining of brain tissue (scale: 500 μm and 100 μm, Black arrows represent neuronal pyknosis). (**D**) The number of Nissl-body-positive cells. (**C**,**E**–**H**) Representative blot images and quantification analysis of the expression of Aβ_1–42_, P-Tau, NF-L, and GFAP in the brain. The data are mean ± SEM (*n* = 6 per group). *, # *p* < 0.05; **, ## *p* < 0.01 (* vs. normal group and # vs. model group, respectively).

The potential effects of SCEP treatment on the pathological hallmarks of AD [25], including Aβ, P-Tau, GFAP, and NFL protein and histopathology, were then evaluated. H&E staining of the brain sections was performed after sacrifice to detect pathological changes in each group of mice. As shown by the histopathological analysis, neuron injury was ameliorated via reduced neuronal solidification in the hippocampal DG area, cell volume, irregular shape and arrangement, cytoplasmic demarcation in the nucleus, and cytoplasm in the SCEP intervention group compared with the model group (Figure 2A). Nissl staining further indicated that SCEP could attenuate neuronal damage in AD mice (Figure 2B). When compared with the control group, the protein expressions of Aβ_1–42_ (*p* < 0.01), P-Tau (*p* < 0.05), NF-L (*p* < 0.01), and GFAP (*p* < 0.01) were significantly accentuated in the model group induced by AlCl_3_/D-gal. SCEP gavage treatment at a dose of 300 mg/kg/d effectively reduced the protein levels of Aβ_1–42_ (*p* < 0.05), P-Tau (*p* < 0.01), NF-L (*p* < 0.01), and GFAP (*p* < 0.05) in the brain compared with the model group, as shown in Figure 2C–G. These results demonstrate that the gavage administration of SCEP could reduce neuron damage and astrocyte activation and relieve the pathology of AD, suggesting that SCEP have neuroprotective effects and slow down the progression of AD.

### 3.3. Effects of Gavage Administration of SCEP on Gut Dysbiosis in AD Model Mice

**Figure 3:**
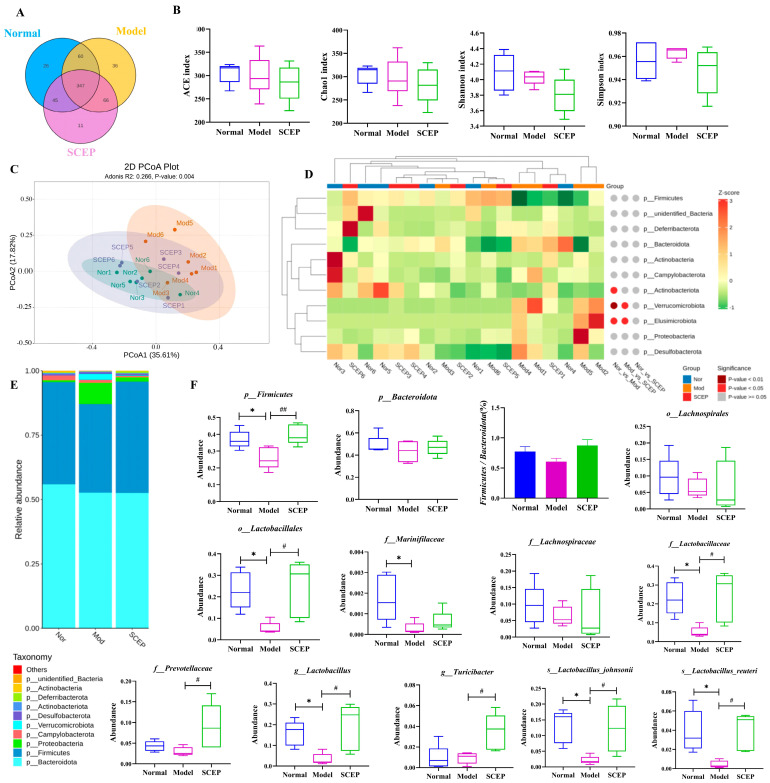
The effects of the gavage administration of SCEP on gut dysbiosis in the AD mouse model. (**A**) Venn diagram. (**B**) The ACE index, Chao1 index, Shannon index, and Simpson index were used to evaluate the richness and evenness of intestinal microorganisms. (**C**) PCoA principal coordinate analysis. (**D**) The heat map of species abundance at the level of phylum. (**E**) A stacked bar chart of the relative abundance of species in different groups at the phylum level. (**F**) A comparison of bacterial abundance at different levels. The data are mean ± SEM (*n* = 6 per group). *, # *p* < 0.05; ## *p* < 0.01; (* vs. normal group and # vs. model group, respectively).

It has been reported that prickly ginseng oligopeptides effectively alleviate hyperuricemia in mice in a microbiota-dependent manner [26]. In this case, we put our sights on the effects of SCEPs on gut dysbiosis. Fresh feces were collected from all groups of mice for 16S rRNA sequencing. As shown in Figure 3A, 26 and 36 specific ASVs were identified in the normal and model groups, respectively, while the SCEP treatment group contained 11 specific ASVs. Alpha-diversity analysis results showed that the AD model mice had no more significant sequencing depth index and bacterial richness than control mice. No differences in the Shannon, Chao1, ACE, and Simpson indices were found between the SCEP (300 mg/kg/d) treatment group and the model group (Figure 3B). However, PCoA showed differences in the intestinal bacterial profiling among the three groups. The samples in the normal and SCEP groups tended to be aggregated with a tendency to move away from the model group, as shown in Figure 3C.

Thus, based on the results of species annotation, the gut microbiota abundance in each group was analyzed at different levels. As shown in Figure 3D–F, at the phylum level, the proportion of *Firmicutes* accounted for 45%, 24%, and 37%, respectively, in the control, model, and SCEP treatment groups. Compared with the normal group, the abundance of *Firmicutes* was significantly reduced in the model group (*p* < 0.05). Notably, SCEP treatment effectively reversed this reduction, as evidenced by the significant increase in the abundance of *Firmicutes* in the SCEP-treated group (*p* < 0.01). Additionally, the *Firmicutes/Bacteroidetes* (F/B) ratio is widely recognized as an indicator of intestinal microbial dysbiosis [27]. Our analysis revealed that the F/B ratio was significantly increased in the SCEP group compared to the model group. At the family level, when compared with the control group, the relative abundances of *Muribaculaceae* (*p* < 0.05), *Lachnospiraceae* (*p* > 0.05), *Lactobacillaceae* (*p* < 0.05), and *Prevoteaceae* (*p* > 0.05) in the model group were significantly decreased by 83%, 34%, 78%, and 30%. However, compared with the model group, SCEP treatment reversed the decreases in the abundances of these bacterial families, with the abundances of *Lactobacillaceae* and *Prevoteaceae* (*p* < 0.05 for both) increasing significantly. At the genus level, the bacterial composition of mice is mainly composed of *Lactobacillus*, *Bacteroides*, *Parabacteroides*, *Turicibacter*, and *Lachnospiraceae*. The relative abundances of *Turicibacter* (*p* > 0.05) and *Lactobacillus* (*p* < 0.05) were attenuated in model mice compared with the control group, while SCEP treatment increased the relative abundances of *Turicibacter* and *Lactobacillus* by 2.7-fold and 4.8-fold compared with the model group.

### 3.4. FMT from the SCEP Treatment Mice Also Attenuated Spatial Memory, Cognitive Deficits, and Pathology in the AD Mouse Model

**Figure 4:**
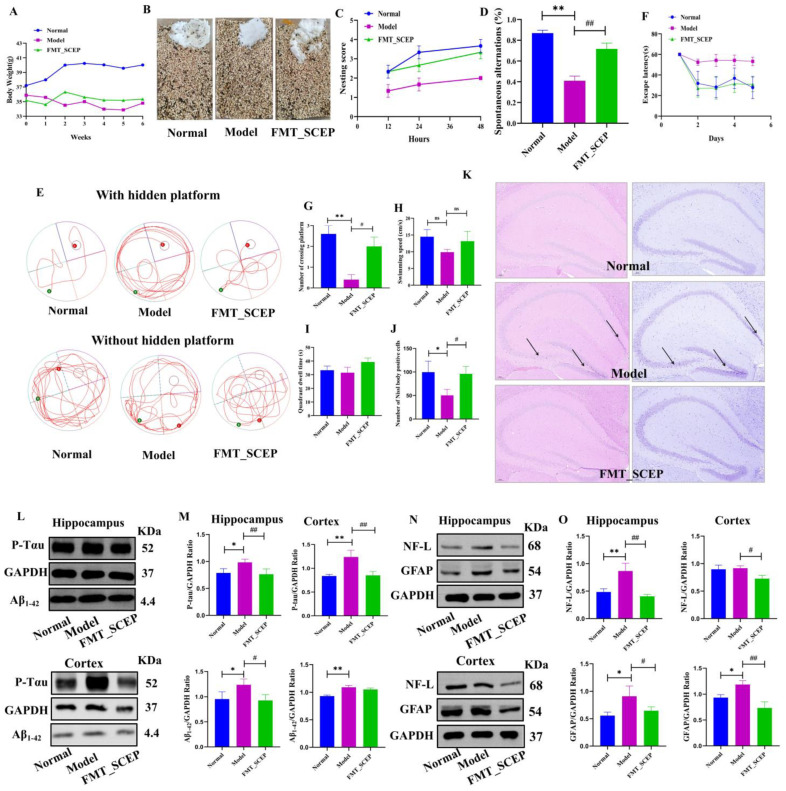
FMT from the SCEP treatment mice also attenuated spatial memory, cognitive deficits, and pathology in the AD mouse model. (**A**) Body weight of mice. (**B**) Individual nesting test’s photographs and (**C**) analysis. (**D**) Y-maze experiment analysis. (**E**) Representative traces in MWM tests (green dot: starting position; red dot: end position). (**F**) Escape latency during training. (**G**) Number of platform crossings. (**H**) Average swimming speed. (**I**) Target quadrant dwell time. (**J**) The number of Nissl-body-positive cells. (**K**) H&E staining and Nissl staining of brain tissue (scale: 500 μm and 100 μm, Black arrows represent neuronal pyknosis). (**L**–**O**) Representative blot images and quantification analysis of the expression of Aβ_1–42_, P-Tau, GFAP, and NF-L in the hippocampus and the cortex, respectively. Data are mean ± SEM (*n* = 6 per group). ns represents no significant difference; *, # *p* < 0.05; **, ## *p* < 0.01 (* vs. normal group and # vs. model group, respectively).

Considering that SCEP was administered to mice via oral gavage and that SCEP may act on the gut microbiota and have neuroprotective effects, the fecal microbiota from the SCEP treatment group mice were given to another batch of AD model mice via gavage (defined as the FMT_SCEP group) to investigate whether gut microbiota mediated neuroprotective effects. As shown in Figure 4A, the average body weight of mice in the model group showed a downward trend during the experiment, while the normal and FMT_SCEP groups showed upward trends. The weights of mice in the model group were generally lower than those in the other two groups. In addition, individual nesting tests showed that compared with the model group, the FMT_SCEP group mice began to nest within 12 h and formed a relatively complete nest within 24 h. The score was significantly higher than that of the model group (*p* < 0.01). The nesting behavior was characterized by the tearing and aggregation of cotton pieces to form a formed and flat nest (Figure 4B,C). Y-maze results showed that the FMT_SCEP group showed an increased spontaneous alternation rate for AD mice compared with the model group (*p* < 0.01; d = 0.820) (Figure 4D). In the MWM test, the escape latency of the FMT_SCEP group decreased, the number of platform crossings increased (*p* < 0.01; d = 0.171), and the target quadrant residence time increased (Figure 4E–I). Similarly, H&E staining and Nissl staining were also conducted. FMT_SCEP treatment reduced the pyknosis of neurons, with more regular shapes and arrangements and clearer nuclear–cytoplasmic boundaries than in the model group (Figure 4J).

Furthermore, pathological hallmark proteins of the cortex and hippocampus were detected separately. It was found that the levels of Aβ_1–42_, P-Tau, NF-L, and GFAP in the hippocampus in the FMT_SCEP group were also markedly attenuated (*p* < 0.05, *p* < 0.05, *p* < 0.01, *p* < 0.05) compared with the model group. The trends were consistent with those in the SCEP treatment experiments, as shown in Figure 2, indicating that FMT_SCEP treatment reversed the alteration of the elevated hallmark proteins in the model group. Interestingly, the levels of Aβ_1–42_, P-Tau, NF-L, and GFAP in the cortex showed some different alterations, as shown in Figure 4K–N. Overall, the FMT_SCEP treatment could significantly reduce the levels of P-Tau, NF-L, and GFAP in the cortex compared with the model group, but no effect on Aβ_1–42_ was observed. These results validate the hypothesis that SCEPs are mediated by the gut microbiota to produce neuroprotective effects.

### 3.5. Comparison of the Similarity of Mice Gut Microbiota Profiling After SCEP and FMT_SCEP Treatment

**Figure 5:**
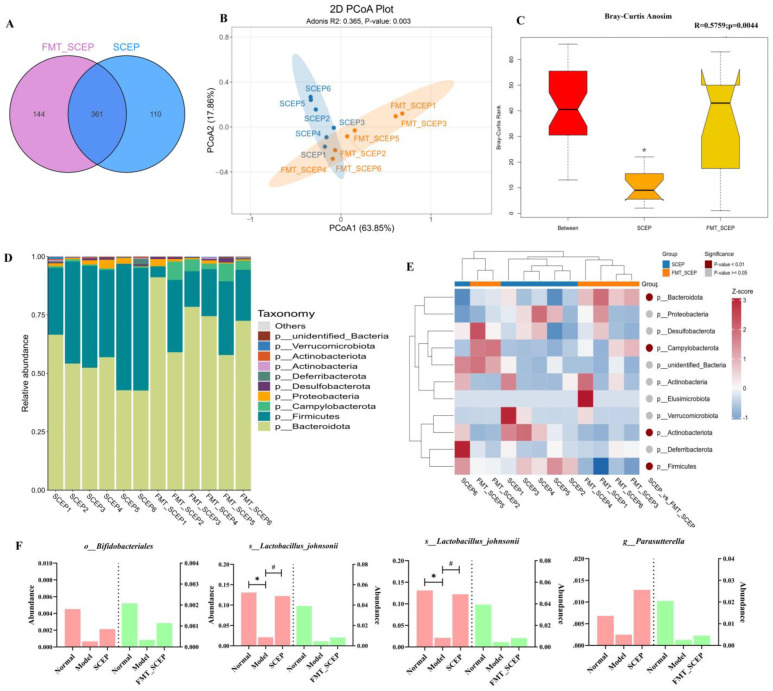
A comparison of the similarity of mice gut microbiota profiling after SCEP and FMT_SCEP treatments. (**A**) Venn diagram. (**B**) PCoA principal coordinate analysis. (**C**) Anosim group difference analysis chart based on ASV. (**D**) A stacked bar chart of the relative abundance of species at the level of phylum. (**E**) A heat map of species abundance at the phylum level. (**F**) Similarity of abundance changes at different classification levels (species, genus, order) of gut bacteria. The data are mean ± SEM (*n* = 6 per group). *, # *p* < 0.05; (* vs. normal group and # vs. model group, respectively).

We compared the similarity of gut bacteria changes between the SCEP and FMT_SCEP groups. There were 361 identical ASVs in the two groups. Moreover, 110 and 114 specific ASVs were identified in the SCEP group and the FMT_SCEP group, respectively (Figure 5A). Here, the FMT treatment group had more ASVs under the condition of low-load intestinal microbiota due to antibiotic pretreatment, suggesting that FMT treatment may be more conducive to colonizing some intestinal microbiota. As shown in Figure 5B, PCoA demonstrated the obvious clustering of samples between the two groups. The ASV-based Anosim group difference analysis of the SCEP and FMT_SCEP group samples showed that the R-value was greater than 0 (R = 0.5759) (Figure 5C), indicating that the difference between them was still significant. Based on the results of species annotation, we conducted an in-depth screening of intestinal bacteria with similar alteration trends between the SCEP and FMT_SCEP treatment groups. It was found that at the phylum level, the intestinal bacteria of both groups of mice were mainly composed of *Bacteroidota*, *Firmicutes*, *Campylobacterota*, *Proteobacteria*, and *Deferribacteres* (Figure 5D,E). Four intestinal bacteria were obtained at the three levels of order, species, and genus. They were *o_Bifidobacteriales*, *g_Parasutterella*, *s_Lactobacillus_reuteri*, and *s_Lactobacillus_johnsonii*, the abundances of which decreased in the model group compared with the normal group, while the two treatment groups of SCEP and FMT_SCEP both reversed this change in AD mice (Figure 5F). The four mentioned intestinal bacteria were speculated to be key bacteria in gut microbiota-mediated SCEPs, alleviating cognitive impairment in AD mice. Moreover, we note that these four bacteria are typical of SCFA-producing bacteria.

### 3.6. Gavage Administration of SCEP Improved the Levels and Distribution of Gut Microbial Metabolite SCFAs and Regulated the Transporter of MCT-1

**Figure 6:**
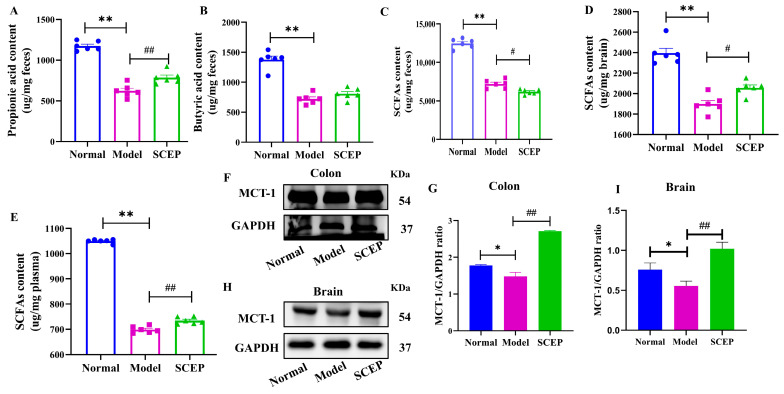
The gavage administration of SCEP improved the levels and distribution of gut microbial metabolite SCFAs and regulated the transporter of MCT-1. The levels of (**A**) propionic acid, (**B**) butyric acid, and (**C**) total SCFAs in feces. (**D**) The levels of total SCFAs in blood. (**E**) The levels of total SCFAs in the brain. (**F**–**I**) Representative blot images and quantification analysis of MCT-1 in colon and brain. The data are mean ± SEM (*n* = 6 per group). *, # *p* < 0.05; **, ## *p* < 0.01 (* vs. normal group and # vs. model group, respectively).

To further examine the changes in the levels and distribution of metabolite SCFAs from key bacteria, we quantified the levels of SCFAs in feces, blood, and brain tissue samples of mice in each group using LC-MS. Compared with the normal group, the model group had lower levels of total SCFAs in feces (*p* < 0.01; d = 0.989), while SCEP treatment resulted in increased levels of propionic acid (*p* < 0.01; d = 0.978) and butyric acid, and no significance was found for total SCFAs in the feces of AD mice (Figure 6A–C), suggesting that SCFA-producing bacteria promote the levels of propionic acid and butyric acid in the intestine. Compared with the normal group, the total SCFA levels in the blood and brain of mice in the model group decreased (*p* < 0.01 for both; d = 0.993; d = 0.835), while SCEP treatment reversed the downward trend of total SCFA content in AD mice, and the levels of total SCFAs increased compared with the model group (Figure 6D,E), indicating that SCEP treatment induced alterations in the distribution and levels of SCFAs in feces, blood, and brain tissues by remodeling the SCFA-producing microbiota in the intestine.

To explore the transport mechanism of SCFAs, we detected the expression levels of MCT-1 in the brain tissue and colon of mice in each group. As shown in Figure 6F–I, in both the colon and the brain, the expression levels of MCT-1 in the model group significantly decreased compared with the normal group (*p* < 0.05 and *p* < 0.05), while SCEP treatment significantly increased the levels of MCT-1 in AlCl_3_/D-gal-induced AD model mice (*p* < 0.01 and *p* < 0.01). These results suggest that MCT-1 induced the re-distribution of SCFAs, which may mediate the protective effect of SCEP on gut–brain barrier dysfunction in AD model mice.

### 3.7. Gavage Administration of SCEPs Improved the Intestinal and Blood–Brain Barrier Function of AD Mice

The intestinal epithelial barrier, or blood–brain barrier (BBB), is a physical and functional barrier composed of a tight junction between a single layer of epithelial cells [28]. When the integrity of the barrier is damaged, toxins and other substances may translocate and re-distribute between tissue and the bloodstream [29]. Thus, maintaining the integrity of the barrier is crucial for tissue homeostasis and health. Tight junction (TJ) proteins such as claudins, ZO-1, and occludins contribute to barrier function and control the entry and exit of substances [30].

**Figure 7:**
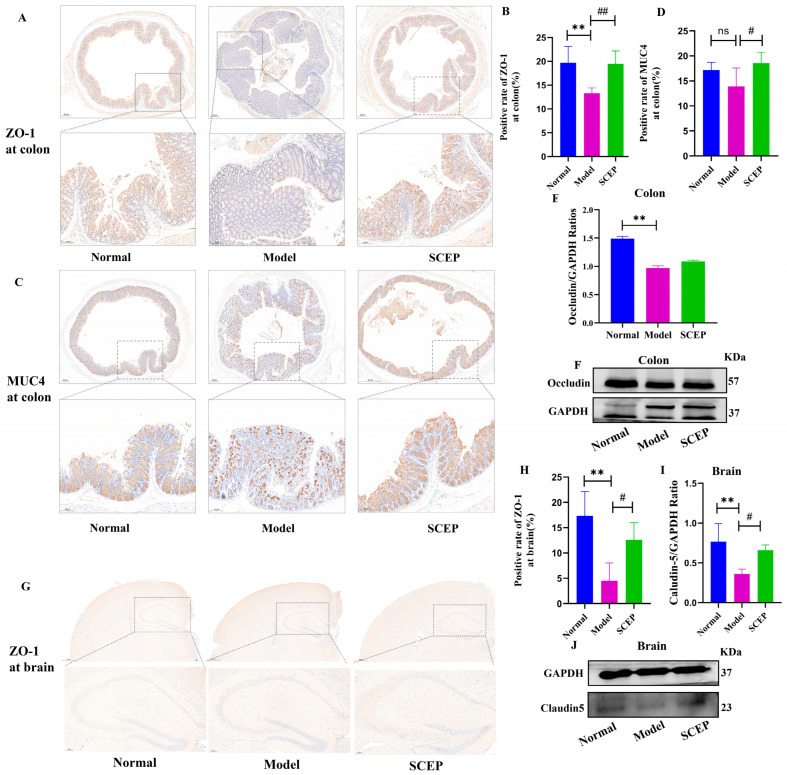
The gavage administration of SCEP improves intestinal and blood–brain barrier function of the AD mouse model. (**A**–**D**) Representative immunofluorescence staining and quantification of fluorescent intensity of ZO-1 and MUC4 in the colon. (**E**,**F**) Representative blot images and quantification analysis of occludin in the colon. (**G**,**H**) Representative immunofluorescence staining and quantification of fluorescent intensity of ZO-1. (**I**,**J**) Representative blot images and quantification analysis of claudin5 in the brain. The data are mean ± SEM (*n* = 6 per group). ns represents no significant difference; # *p* < 0.05; **, ## *p* < 0.01 (* vs. normal group and # vs. model group, respectively).

Therefore, we examined the TJ proteins in the colon and brain. Immunohistochemistry and Western blot results are shown in Figure 7. Compared with the normal group, the colon expression of ZO-1 and occludin (*p* < 0.01 for both) in the model group was significantly decreased. The decreased expression of the mucus barrier protein MUC4, the key regulatory target of gut barrier and homeostasis, was also found in the colon tissue but with no significance (*p* > 0.05). Compared with the model group, in most instances, SCEP significantly upregulated the expression of ZO-1 (*p* < 0.01), occludin (*p* > 0.05), and MUC4 (*p* < 0.05) in the colon (Figure 7A–F), thereby minimizing intestinal injury and strengthening the mucus barrier. Furthermore, AlCl_3_/D-gal induced the downregulation of the brain expression of ZO-1 and claudin5 (*p* < 0.01 for both) compared with the normal group, while SCEP treatment reversed the downtrend in model mice (*p* < 0.05 for ZO-1, *p* < 0.05 for claudin5) (Figure 7G–J), indicating that SCEP alleviated BBB dysfunction in AD model mice. These results suggest that SCEP can significantly improve the integrity of the intestinal barrier and the blood–brain barrier, reducing their permeability, thereby restoring barrier function.

### 3.8. Gavage Administration of SCEP Suppressed HDAC3 Expression, in Turn Upregulating BDNF and NT3 Levels in the AD Mouse Model

**Figure 8:**
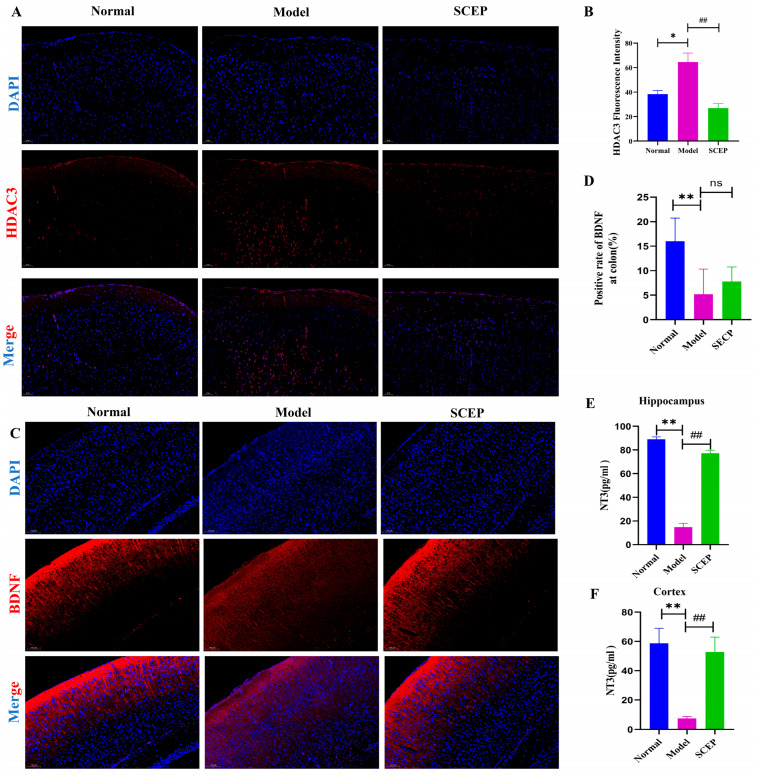
The gavage administration of SCEPs suppressed the HDAC3 pathway and upregulated BDNF and NT3 levels in the AD mouse model. (**A**,**B**) Representative immunofluorescence staining and quantification of the fluorescent intensity of HDAC3 and DAPI in different groups. (**C**,**D**) Representative immunofluorescence staining and quantification of the fluorescent intensity of BDNF and DAPI in different groups. (**E**,**F**) The quantification of NT3 protein levels at the hippocampus and cortex via ELISA. The data are mean ± SEM (*n* = 6 per group). ns represents no significant difference; * *p* < 0.05; **, ## *p* < 0.01 (* vs. normal group and # vs. model group, respectively).

Histone deacetylases (HDACs) have recently garnered attention as an interesting target for neurodegenerative diseases therapies, since HDAC inhibitors can reinstate memory even after neuronal loss [31,32,33,34]. In this study, the expression of HDAC3 in mouse brain tissue was determined via both immunofluorescence and Western blotting. Compared with the normal group, the expression of HDAC3 in the model group was significantly increased, consistent with clinical sample studies [35]. Promisingly, SCEP treatment significantly reduced the level of HDAC3 protein in the brains of the model mice (*p* < 0.01) (Figure 8A,B). To investigate the effects of SCEP treatment on the neurotrophic factors, immunofluorescence and ELISAs were performed to assess the expression of BDNF and NT3. In the model group, quantitative analysis revealed significant decreases in cortical BDNF and NT3 levels compared with the control group, emphasizing the low neurotrophic state of neurons in the AD model mice, while SCEP treatment upregulated the levels of BDNF (*p* > 0.05) and NT3 (*p* < 0.01 at both the hippocampus and cortex) proteins in the brains of model mice (Figure 8C–F). The results show that SCEPs could restore the overexpression of HDAC3 and recover the downregulation of BDNF- and NT3-associated signaling in AD mice.

## 4. Discussion

AlCL_3_ and D-gal co-administration induces cognitive decline in mice, establishing a representative AD model [36]. D-gal promotes reactive oxygen species accumulation and accelerates the formation of advanced glycation end products, ultimately leading to oxidative stress, effectively mimicking natural aging [37]. Aluminum exposure, a recognized environmental risk factor for AD, promotes the aberrant cleavage of the amyloid precursor protein, increasing the generation and deposition of both Aβ_40_ and Aβ_42_ peptides. Concurrently, it induces tau protein phosphorylation, leading to neurofibrillary tangle formation [38]. In this study, AlCl_3_/D-gal-induced AD mouse models showed typical pathological and behavioral characteristics of AD, including abnormalities in cognitive behavior, cognitive dysfunction, and pathological hallmarks of AD, including Aβ, P-Tau, GFAP, and NF-L protein and histopathology in brain tissue. These manifestations are consistent with previous research results [39].

Based on the characteristics of high protein and low fat and the varied biological activities of sea cucumber, ever-more researchers are paying attention to the preparation and biological activity of sea cucumber peptides. Sea cucumber peptides are a protein hydrolysate obtained via separating and purifying sea cucumber after protease hydrolysis. Sea cucumber peptides have good solubility, stability, and low viscosity. They are not easily destroyed by proteases and acid–base substances in the digestive system, so they can be fully absorbed and utilized by the human body [40]. Studies have shown that sea cucumber peptides can improve memory by repairing neuronal cells in hippocampal CA1 and CA3 regions and enhancing the number of Nissl bodies [41]. Zhao et al. [42] proved that sea cucumber peptides can alleviate scopolamine-induced cognitive impairment by regulating oxidative imbalance, reducing cholinergic dysfunction and improving nerve injury. In this study, SCEP definitely improved cognitive deficits and reduced Aβ_1–42_, P-Tau, NF-L, and GFAP protein expression in the brain tissues of AD mice, with the potential to alleviate AD development.

The microbiota–gut–brain axis is the complex bidirectional communication network between gut bacteria and the brain, essential for maintaining the homeostasis of the gastrointestinal, central nervous, and microbial systems [43]. Studies have shown that the disruption of the axis is associated with varied neurodegenerative disorders [44], of which both AD and Parkinson’s disease (PD) may be associated with changes in the levels of specific SCFAs and SCFA-producing bacteria [45]. For example, in AD model mice, the diversity of the gut microbiota was shown to be altered, including reduced abundances of butyric acid producers, similar to the results of human studies [46,47]. Furthermore, the concentrations of SCFAs (including propionic acid and butyric acid) appeared to be lower in AD model mice than in wild-type mice [46,48]. Sun et al. [49] demonstrated that transplanting the fecal microbiota could ameliorate many pathological features in APP/PS1 mice, accompanied by improvements in gut microbiota and SCFAs, as well as that the protective effect of fecal microbiota transplantation may be associated with the reversal of SCFA alterations in AD mice.

In this study, we found that SCEP increased the *Firmicides/Bacteroides* ratio and decreased the abundance of *Proteobacteria* in model mice. The F/B ratio has an important impact on maintaining intestinal homeostasis [50]. *Proteobacteria* is a low-abundance bacterium in healthy intestines, and its increased abundance is generally considered a potential diagnostic criterion for microbial dysfunction and disease risk [51]. Meanwhile, AlCl_3_/D-gal decreased the abundances of relevant SCFA-producing bacteria in the intestines of AD mice, in turn leading to decreases in the content of SCFAs in the intestinal contents of AD mice. SCEP gavage also increased the abundances of *Lactobacillaceae*, *Prevotellaceae*, *Turicibacter*, and *Lactobacillus*. Importantly, four SCFA-producing bacteria in the gut were obtained through comparisons of gut microbiota profiling between SCEP treatment and FMT treatment, which plays a key role in reversing the decrease in the content of SCFAs in the colon of AD mice. The results of the fecal microbiota transplantation experiment further support the idea that SCEPs alleviate the development of AD by modifying the profiling of the intestinal bacteria in AD mice.

Studies have shown that SCFAs are produced by various bacteria common in the intestines, such as *Bifidobacterium*, *Lactobacillus*, *Bacteroides*, and *Firmicutes* [52,53]. A small amount of SCFAs come from food and the host metabolism. In vivo, SCFAs are first absorbed and metabolized by colon cells, and unmetabolized SCFAs enter the portal vein circulation. In the liver, SCFAs are further metabolized, and only a small amount enters systemic circulation and reaches the peripheral tissue. The circulation level and availability of SCFAs largely depend on the intake of dietary fiber and the number of SCFA-producing bacteria in the colon [54,55]. Butyrate-producing bacteria upregulate MCT-1 expression by activating SCFA receptors, thereby promoting intestinal stability and bacterial diversity [56]. A study on the SCFAs in vivo showed that the treatment of SCEP induced the increased expression of MCT-1 in both the colon and the cortex, resulting in the re-distribution and increased levels of SCFAs in the feces, blood, and brain of AD mice, which may help to mediate the neuroprotective effect of SCEP.

Relevant studies have demonstrated that the increase in barrier permeability induced by varied pathological stimuli leads to leukocyte infiltration, the influx of water and plasma proteins, the passage of bacteria and their virulence products, and the entry of pro-inflammatory mediators, which in turn cause glial cell activation, inflammation, and neuronal dysfunction [57]. Many neurological disorders associated with aging, neuroinflammation, and neurodegeneration, such as AD, PD, and behavioral and psychiatric disorders, are accompanied or even primarily caused by the leakage of the blood–brain barrier [58,59]. TJ proteins, especially tight proteins, occludins, and junctional adhesion molecules, interact with their corresponding proteins on neighboring endothelial cells. In this study, we found that the associated TJ protein abundance was decreased in the brain tissues of AD mice, which indicated that the induction of AlCl_3_/D-gal opened the BBB in AD mice, whereas the treatment of SCEP upregulated the abundance of TJ proteins. The tight junction protein ZO-1 was critical for effective mucosal repair [60] at the BBB, and claudin-5 is thought to be the dominant TJ protein [61]. SCEP intervention not only enhanced the expression of TJ proteins and improved cellular connectivity but also decreased the permeability of the gut and the blood–brain barrier, suggesting that SCEP could improve the BBB.

In this study, we showed that SCEP suppressed HDAC3 expression and enhanced neurotrophic factor BDNF expression and NT3 expression in the brain tissue. Previous studies have shown that nonselective class I HDAC inhibitors increase BDNF expression and learning and memory, and some of these effects are mediated by HDAC2 and HDAC3 [62,63]. For example, Sartor et al. found that HDAC2 and HDAC3 are important negative regulators of BDNF expression [64]. The benefits of NT3 were reported in treating memory deficits, indicating its potential in inherited and acquired forms of dementias [65], which revealed that the pharmacological inhibition of HDAC3 increased BDNF expression [66] and the potential role of NT3 in AD treatment using an NT3-transduced graft [67]. Inhibiting the activity or expression of HDAC3 can significantly reduce the increases in permeability and downregulation of TJ protein induced by different pathological stimuli and protect the BBB from damage [68,69]. SCFAs inhibit HDAC activity and can reduce HDAC expression in cells [70]. In this study, it was concluded that SCEP inhibited HDAC3 expression, and it was speculated that the increase in microbial-derived SCFAs was involved in the regulation, thereby restoring the increased blood–brain barrier permeability and downregulated TJ protein in the AD model, protecting the BBB from damage.

**Figure 9:**
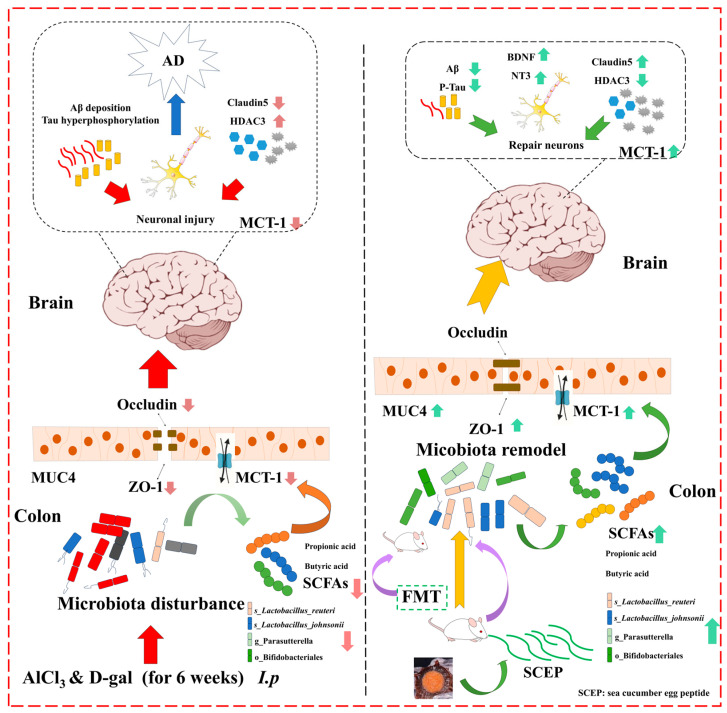
Potential mechanism of SCEP ameliorates cognitive impairments and reduces AD pathology.

Taken together, in this study, we prepared the low-molecular-weight oligopeptide SCEP from sea cucumber egg via simulated gastrointestinal digestion. As shown in Figure 9, we found that SCEP significantly improved the cognitive and memory impairment of the AD mouse model and reduced AD pathology by suppressing HDAC3, in turn increasing the expression of BDNF and NT3 via gut microbiota and SCFAs, suggesting that SCEP can slow down the progression of AD. However, there are still some limitations to our study. The SCEPs obtained were of an oligopeptide mixture, and the material basis for the production of function still needs to be further isolated and identified. In addition, molecular dynamics simulations may be considered in subsequent studies to find relevant targets. Finally, although we analyzed the HDAC3 pathway, we have not yet found or identified the upstream and downstream targets of HDAC3. Thus, this study provides a basis for considering SCEP as a promising candidate for neuroprotection and a reference point for developing and applying sea cucumber eggs.

## 5. Conclusions

In summary, the present study systematically elucidated the mechanism by which sea cucumber intestinal epithelial peptides (SCEPs) improve memory in AlCl_3_/D-gal induced memory-impaired mice. Selected as key study subjects due to their notable performance in behavioral tests, SCEPs were found to potentially alleviate neuroinflammation and reduce neuronal damage through remodeling of the gut microbiota in mice. This includes improving dysbiosis of the intestinal flora and modulating the content and distribution of its metabolites, such as short-chain fatty acids. Furthermore, SCEPs may regulate the HDAC3 pathway, thereby enhancing the expression of NT3 and BDNF, and repairing impairments in both the intestinal barrier and blood-brain barrier. These findings suggest that SCEPs could provide novel insights into the development of functional foods for individuals with memory impairment. However, further research is still needed to clarify the specific brain targets of SCEPs and the detailed molecular mechanisms underlying their effects on memory improvement in vivo.

## Data Availability

Data will be made available on request.

## Supplementary Materials

The following supporting information can be downloaded at https://www.mdpi.com/article/10.3390/nu17142312/s1, Table S1: SCEP timing planning and design; Table S2: FMT timing planning and design; Table S3: Scoring rules for nesting experiments.

## Abbreviations

SCEP	Sea cucumber eggs oligopeptides
AD	Alzheimer’s disease
FMT	Fecal microbiota transplantation
AlCl_3_	Aluminum chloride
D-gal	D-galactose
Aβ	β-Amyloid
P-Tau	Phosphorylated tau protein
SCFAs	Short-chain fatty acids
BCA	Bicinchoninic Acid Assay
TJ	Tight junctions
CNS	Central nervous system
MWM	Morris water maze
HE	Hematoxylin–eosin staining
ZO-1	Right junction protein 1
claudin 5	Compact linking protein 5
HDAC3	Histone deacetylase 3
GFAP	Glial fibrillary acidic protein
NF-L	Neurofilament light chain
MCT-1	Monocarboxylate transporter-1
MUC4	Mucin-4
LC-MS	Liquid chromatography–mass spectrometry
PVDF	Polyvinylidene difluoride
GAPDH	Glyceraldehyde-3-phosphate dehydrogenase
DG	Dentate gyrus
BBB	Blood–brain barrier
NT3	Neurotrophin-3
BDNF	Brain-derived neurotrophic factor
DAPI	4′,6-Diamidino-2′-phenylindole
PD	Parkinson’s disease
