# Supplementary material for: Sea Cucumber Egg Oligopeptides Ameliorate Cognitive Impairments and Pathology of Alzheimer’s Disease Through Regulating HDAC3 and BDNF/NT3 via the Microbiota–Gut–Brain Axis
Source: Nutrients. 2025 Jul 14;17(14):2312. doi: 10.3390/nu17142312 (PMC12298608; doi:10.3390/nu17142312)
Supplement: Supplementary file 1 [file nutrients-17-02312-s001.zip › nutrients-3702000-supplementary.pdf]

Table.S1 SCEP timing planning and design.

Days	Normal	Model	SCEP	
0d	SPSS i.p	$Alcl_3$+D-gal i.p	SCEP i.g	$Alcl_3$+D-gal i.p
7d	SPSS i.p	$Alcl_3$+D-gal i.p	SCEP i.g	$Alcl_3$+D-gal i.p
14d	SPSS i.p	$Alcl_3$+D-gal i.p	SCEP i.g	$Alcl_3$+D-gal i.p
21d	SPSS i.p	$Alcl_3$+D-gal i.p	SCEP i.g	$Alcl_3$+D-gal i.p
28d	SPSS i.p	$Alcl_3$+D-gal i.p	SCEP i.g	$Alcl_3$+D-gal i.p
36d	SPSS i.p	$Alcl_3$+D-gal i.p	SCEP i.g	$Alcl_3$+D-gal i.p
42d	Sample Collection	Sample Collection	Sample Collection	

Table.S2 FMT timing planning and design.

Days	Normal	Model	FMT
0d	SPSS i.p	$Alcl_3$+D-gal i.p	AMP+GM+MTZ i.p
7d	SPSS i.p	$Alcl_3$+D-gal i.p	Bacterial solution i.p
14d	SPSS i.p	$Alcl_3$+D-gal i.p	Bacterial solution i.p
21d	SPSS i.p	$Alcl_3$+D-gal i.p	Bacterial solution i.p
28d	SPSS i.p	$Alcl_3$+D-gal i.p	Bacterial solution i.p
36d	SPSS i.p	$Alcl_3$+D-gal i.p	Bacterial solution i.p
42d	Sample Collection	Sample Collection	Sample Collection

Table.S3 Scoring rules for nesting experiments.

Score	Nesting situation
1	Cotton flakes scattered in cage, no visible nests, no visible bite marks.
2	Cotton pieces loosely stacked on the side of the cage, no visible nests, no visible bite marks.
3	Cotton flakes are aggregated to form a shaped but flat nest with no visible tearing.
4	Cotton flakes are torn and gathered to form a shaped but flat nest.
5	Cotton flakes were apparently torn and gathered by the animal and piled up to form a deep nest.
